# Increased accuracy of starch granule type quantification using mixture distributions

**DOI:** 10.1186/s13007-017-0259-2

**Published:** 2017-12-06

**Authors:** Emi Tanaka, Jean-Phillippe F. Ral, Sean Li, Raj Gaire, Colin R. Cavanagh, Brian R. Cullis, Alex Whan

**Affiliations:** 1 School of Mathematics and Applied Statistics, University of Wollongong, Northfields Ave, Wollongong, 2522 Australia; 2 CSIRO Agriculture and Food, 1600 Clunies Ross Street, Canberra, 2601 Australia; 3 Bayer CropScience Pty Ltd, Seeds, Technologiepark 38, 9052 Zwijnaarde (Gent), Belgium; 4 School of Mathematics and Statistics, University of Sydney, Carslaw F07, Sydney, 2006 Australia

**Keywords:** Starch, Granule type, Mastersizer, Mixture distribution

## Abstract

**Background:**

The proportion of granule types in wheat starch is an important characteristic that can affect its functionality. It is widely accepted that granule types are either large, disc-shaped A-type granules or small, spherical B-type granules. Additionally, there are some reports of the tiny C-type granules. The differences between these granule types are due to its carbohydrate composition and crystallinity which is highly, but not perfectly, correlated with the granule size. A majority of the studies that have considered granule types analyse them based on a size threshold rather than chemical composition. This is understandable due to the expense of separating starch into different types. While the use of a size threshold to classify granule type is a low-cost measure, this results in misclassification. We present an alternative, statistical method to quantify the proportion of granule types by a fit of the mixture distribution, along with an R package, a web based app and a video tutorial for how to use the web app to enable its straightforward application.

**Results:**

Our results show that the reliability of the genotypic effects increase approximately 60% using the proportions of the A-type and B-type granule estimated by the mixture distribution over the standard size-threshold measure. Although there was a marginal drop in reliability for C-type granules. The latter is likely due to the low observed genetic variance for C-type granules.

**Conclusions:**

The determination of the proportion of granule types from size-distribution is better achieved by using the mixing probabilities from the fit of the mixture distribution rather than using a size-threshold.

**Electronic supplementary material:**

The online version of this article (10.1186/s13007-017-0259-2) contains supplementary material, which is available to authorized users.

## Background

Starch derived from the endosperm of cereal grains is the major source of carbohydrate in the human diet and has many functional uses in food and non-food industries. Starch is made up of two distinct components, amylopectin (70–80% dry biomass) and amylose, which are organised into water-insoluble granules during synthesis (for a review see [1]). Distributions of granule size in cereal endosperm are accepted to be bimodal and classified as large A-type granules and small B-type granules [2–7]. There are reports that verify the existence of an additional type—tiny C-type granules [8–10] but some classify these as B-type. These C-type granules are less studied. A-type granules begin to form in the first week after anthesis. B-type granules form in the second week after anthesis while C-type granules form at the end of the third week after anthesis [10–12].

In starch storage organs, as well as photosynthetic tissue, many enzymes involved in transitory starch biosynthesis have been shown to influence the size of starch granules. These include ADP-glucose pyrophosphorylase [13], starch branching enzyme [14], limit dextrinase [15], starch phosphorylase [16], glucan, water dikinase [17] and starch synthases [18, 19]. In photosynthetic tissue of the model *Arabidopsis thaliana* starch synthase III and IV have been implicated in the initiation of transitory starch granules [20, 21], however, the means by which granules are initiated in cereal endosperm remains unclear. As an essential element of starch production, increased understanding of granule initiation may allow identification of new pathways towards increasing cereal yield.

Perhaps more immediately, an ability to produce cereal starch with optimised starch granule type and size would be of great use in industrial applications of starch. An increased proportion of A-type granules is preferable in many industrial settings, since smaller B-type granules are easily lost during starch isolation. A-type and B-type granules have different chemical and structural properties, notably, A-type and B-type granule have a disc and spherical shapes respectively [7] with different proportions of amylose and amylopectin [22]. These differences give rise to altered functional properties such as an increase in dough elasticity due to a higher proportion of B-type granules [23].

To study the proportion of granule types in a starch sample, it is therefore necessary to be able to discriminate between granule types. A true quantification of granule types would be based on separation of granules based on composition or origin [24]; however, such method is expensive although granule size measurement is relatively straightforward. Before 1990, most studies measured granule sizes with a Coulter Counter, which were unable to accurately measure sizes below 3 μm [8]. More recently granule size distributions have been measured with Mastersizer particle size analysers, or have manually identified granule types using light microscopy.

Granule size distributions of wheat starch show clear multi-modality, either bimodal [4, 5] or trimodal [9]. The differences may be due to the environment, such as water regime, temperature and light intensity [25–29] and in some cases due to a limitation in equipment [8]. Furthermore, differences between cultivars have a major role in influencing starch granule size distribution [2, 30].

**Fig. 1:**
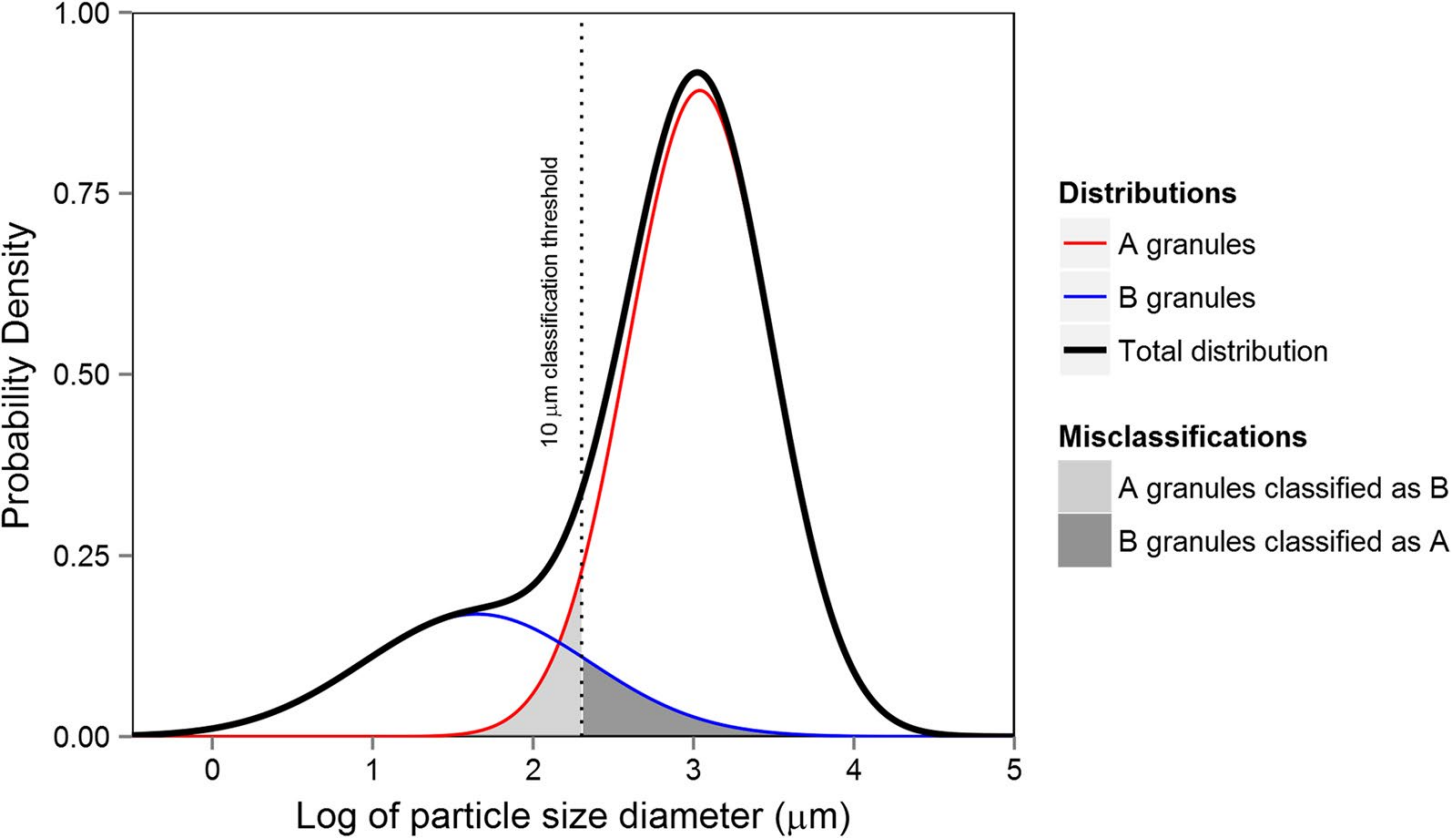
A theoretical distribution of starch granule sizes showing misclassification by size threshold discrimination. Two underlying distributions of A-type (red line) and B-type (blue line) granule types add togethe r to give the observed granule size distribution (black line). Where a size discrimination threshold is used, such as  μm (vertical dotted line), a proportion of granules will be misclassified. In this example, the lower tail of the A-type distribution is misclassified as B-type (light grey shading) and the upper tail of the B-type distribution is misclassified as A-type (dark grey shading). *Note* *x*-axis is the log with base *e* of the diameter of the granule particle

Starch granule types are highly correlated with their size, as such almost all studies use a size-threshold for classification: large, small and tiny granule sizes correspond to A-type, B-type and C-type granules respectively. The threshold used to classify the granule types differs from study to study, however, most use the diameter size of 9.9–10 μm as a cutoff between A-type and B-type granules [2–4, 6, 7, 9] while some [8, 11] use 15–15.9 μm. The boundary between B-type and C-type granules is less well defined with some [8, 11] using 5–5.3 μm and others [9] using 2.8 μm. While this form of classification is straightforward, unless the size distributions of granule types are completely distinct and non-overlapping, misclassification of granule types will occur (see Fig. 1). Such misclassification may be detrimental for the accurate identification of sources of variation for granule type in cereal starch.

We present an alternative method to accurately quantify the proportions of each granule type in a starch suspension by use of a mixture distribution. We show this ‘mixture measure’ provides a significant difference to the ‘size-threshold measure’, and represents an important step forward in accurately estimating granule populations in cereal starch.

## Methods

### Plant material and starch extraction

A field trial of a diverse wheat population was grown in Yanco, NSW, Australia in 2009 as part of the GRDC CSP112 project. Grain from 155 genotypes was milled, and 5 g of flour was weighed into 50 ml screw capped tubes. The flour was treated with 50 ml of 1% (w/v) sodium metabisulphite in 0.05M NaCl for 30 min rotating on an ELMI RM-2L Intelli-mixer (Riga, Latvia) at 40 rpm. The resulting batter was ultra-sonicated in a Soniclean160T (Thebarton, SA, Australia) water bath for 1 minute and passed through 200 μm nylon mesh. The filtrate was sonicated a further 1 min. The sonicated filtrate was then centrifuged at 5100 rpm in a Sigma 4K15 bench top centrifuge and swing out head Nr. 11150. The pellet was resuspended in 20 ml of 90% Percoll (Life Technologies) and centrifuged at 1500 rpm. The upper phases were poured off the primary starch pellet which was washed three times with Milli-Q water. The isolated starch was resuspended in 5 ml of Milli-Q water, frozen and freeze dried for particle size analysis.

### Experimental design

Field, milling, extraction and measurement phases of the experiment were conducted according to *p*-rep designs [31], which were generated using DiGGer [32]. Briefly, in total 864 granule size measurements were taken from 224 extractions from the flour of 155 genotypes. More details are explained in Additional file 1.

### Particle size measurement

The freeze dried starch was suspended in Milli-Q water, briefly vortexed and starch granule size distributions were determined using a Mastersizer 2000 particle size analyser (Malvern, UK), which measures the percentage of the total starch volume for a given diameter size interval.

### Quantification of granule-type population

We compared two approaches to produce summary statistics for the different granule types. The first (size-threshold measure) was the typical method that previous studies have used, and the second (mixture-measure) involved extracting information from fitted mixture distributions. To the best of our knowledge, the statistics from the latter method have not been used previously to characterise starch granule types.

#### Method 1: size-threshold measure

**Fig. 2:**
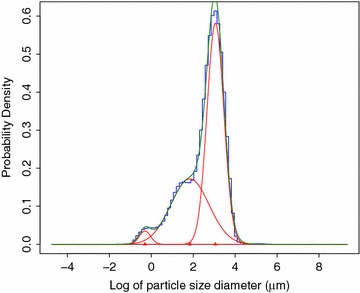
Fit to the particle size distribution across all lines. Particle size distribution was determined from the average total starch volume for each size grouping across all the lines. Red lines are Gaussian density of each individual component. The green line is the mixture density. Blue line is the density histogram of the particle size. *Note* *x*-axis is the log with base *e* of the diameter of the granule particle. The intersection of the red lines is used to derive the size-threshold

To determine suitable thresholds by which to classify the granule types, we fitted a mixture of three Gaussian distribution to the log of the particle size diameter averaged over all samples (Fig. 2). For the threshold between B-type and C-type granules we took the intersection between the fitted individual Gaussian distributions with the smallest and second smallest mean (C-type and B-type granules, respectively). This threshold was 0.969 μm. This cut-off is smaller than previous studies where C-type granules have been considered, which is likely due to differences in environmental conditions, as well as differences between genotypes. The intersection between fits for A-type and B-type granules was at 11.48 μm. Because it is common practice in previous literature to use a threshold of 10 μm, and that was close to the intersection between the distributions we had identified, we chose to use 10 μm as the boundary between A-type and B-type granules. In summary, we used the thresholds (in μm) of $\le 0.954993$, [0.954993, 10) and $\ge 10$ to define C-type, B-type and A-type granules respectively.

#### Method 2: mixture-measure

Because it is the structural and the chemical composition, as well as spatio-temporal origins, that separates the granules into different types rather than size alone, we implemented an alternative method to draw descriptive statistics for the granule types. The plot of the particle diameter size for most genotypes shows a clear trimodal distribution. This distribution appears to be a mixture of three log-normal distributions and thus a mixture of three Gaussian distribution was fitted to the log of particle diameter size for each sample. We interpreted the mixing weights of the three individual component distributions as the proportions of each granule type for the corresponding sample.

**Fig. 3:**
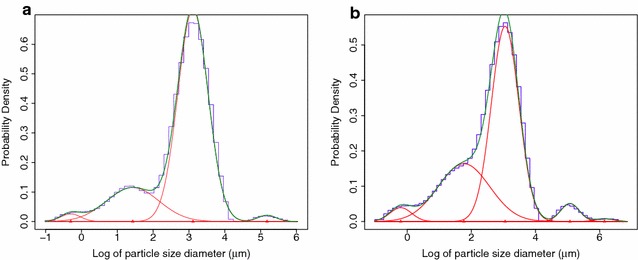
The example fits of the mixture of four or five Gaussian distributions to the log of the particle diameter size

There are several statistical packages available for fitting Gaussian mixture distributions. We chose to use mixdist [33] for the R statistical computing environment [34] because it accepts data input formatted as a frequency table (as is output by the Mastersizer software), and is freely available. An example of the fit from mixdist is given in Fig. 3.

All the fits were checked by visual inspection to assure all the fitted distributions matched the data. Of the 864 particle size measurements, 30 appeared to have a quad-modal distribution and two extraction had a better fit with a mixture of five Gaussian distributions (Fig. 3). These additional distributions were assumed to be due to starch polymerisation or aggregation during measurement. The remaining 96.2% of measurements were well fitted with a mixture of three Gaussian distributions. There were no samples that appeared to have less than three components.

**Fig. 4:**
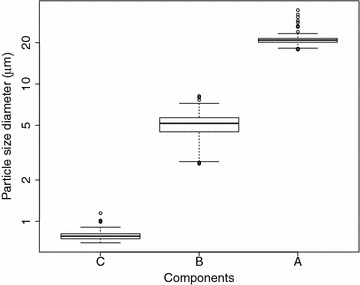
Box plot of the mean of each component of the fitted mixture distribution

The three distributions were assigned as C-type, B-type and A-type granules according to increasing size. Table 1 and Fig. 4 show the five number summary and the box plot of the mean of each component respectively. All components appear to belong to the correct type with no overlap of the means between different types and we observe small variation for the mean of each components. Furthermore, the mean components of A-type and B-type are consistent with previous studies (e.g. [5] reports the peak values of 4.8–6.1 μm for B-type and 21.7–23.9 μm for A-type). We interpreted the mixing weights 100× as the percentage of the total volume for each granule type.

#### Software package

To enable straightforward application of this approach, we have written an R package and web based application, granular. The source code and guide is available at http://doi.org/10.5281/zenodo.344633 and an instance of the web app is hosted at http://shiny.csiro.au/granular/.

The web app allows users to upload starch granule data in a pre-defined format (example data can be found on the site), define peaks interactively and download tabular and graphical output.

**Table 1:** Five number summary of the mean of each component of the fitted mixture distribution

Type	Min	$Q_1$	Median	$Q_3$	Max
A	17.83	20.15	20.87	21.51	34.56
B	2.623	4.490	5.163	5.690	8.156
C	0.6963	0.7454	0.7779	0.8112	1.1440

## Results

Table 2 shows the five number summary of the percentage of the total starch volume for each granule type according to the size-threshold measure and the mixture-measure. Summary from both methods appear close but performing a *t* test for the mean difference of the percentages show that there is a significant difference between the two methods (*p* value $<2.2\times 10^{-16}$). Most samples contain a large volume of A-type (which is consistent with previous studies) and the least volume of C-type.

**Table 2:** Five number summary of the percentage of the total starch volume and mean-genotype reliability for each size-type by method

Method	Type	Min	$Q_1$	Median	$Q_3$	Max	Reliability
Size-threshold	A	49.19	63.59	68.06	74.18	90.09	0.166
	B	8.488	23.370	29.220	33.400	47.900	0.174
	C	0.4684	2.2830	2.6660	2.9720	3.9750	0.293
Mixture-measure	A	40.59	55.82	62.93	73.52	91.59	0.264
	B	6.263	24.210	33.420	40.110	54.020	0.279
	C	0.000009	2.283000	3.118000	3.884000	6.535000	0.036

A more accurate estimate of the phenotypic trait will no doubt improve the accuracy of the genotypic effects should there be any underlying genetic mechanism for the phenotypic trait. The accuracy of the prediction of the genotypic effects can be evaluated by the reliability as defined in [35]. Specifically, we fit a linear mixed model to each estimate of the proportion of the granule-type for the prediction of the genotypic effect taking into account appropriate blocking terms. Reliability is calculated as the squared correlation between the estimated genotypic effect and its true value. Further details of the model and reliability calculation are outlined in Additional file 1. The mean-genotype reliability for each combination of granule type and proportion estimation method is reported in Table 2. We observe a 60% increase in reliability using the mixture measure over the size-threshold measure for A-type and B-type granules, although notably, there is an 88% decrease for C-type granules.

## Discussion

Different composition of the wheat starch gives rise to different utilisation and suitability for various industrial purposes. Wheat starch is composed mostly of A-type and B-type granules in addition to small amounts of C-type granules. The high correlation of these granule types with the particle size makes the use of particle size distribution as a convenient surrogate for the expensive isolation of the granule types. Majority of studies indeed use size distribution to derive further properties about the granule types, however, the classification of granule types is almost exclusively conducted by a simplistic size-threshold method.

We observed that there was typically a tri-modal distribution for the (log of) particle size that was well fitted by a mixture of three Gaussian distribution. The mixing weights associated with the fit of this distribution serves as a better estimation of the proportion of granule types. This was reflected in the observation of a significantly higher reliability for A-type and B-type granules for the mixture measure than the conventional size-threshold measure, although we note this was not the case for C-type granule. The reason for the latter may be due to factors such as the lower machine precision for distribution of smaller particle sizes or the size-threshold measure incorrectly classifying the smaller B-type granules as C-type granules when the B-type granule may be a more highly heritable trait. The latter is indeed supported by the small genetic variance associated for C-type granule as seen in Additional file 1.

One huge disadvantage of the size-threshold measure is that a threshold must be specified for the classification of the granule types. There is no consensus for this threshold across the literature and rather our data suggest variability for an appropriate threshold of each sample, probably owing to different genotype-environment interaction. On the contrary, the mixture-measure does not require specification of a threshold for the classification of the granule types and additionally provides standard errors associated with the estimate of the proportions.

The mixture-measure may be sensitive to the initial values of the parameters and the users should be wary to visually check the fit of the distribution.

## Conclusion

We advocate the use of the mixture-measure over the size-threshold measure as a more accurate estimate of the A-type and B-type granule population in starch samples. Our experimental data shows a significant difference between the two measures and it is clear the size-threshold measure would likely over-estimate or under-estimate the population size of the granule-types with such a rigid classification of the granule-types.

## Additional file

**Additional file 1.** Experimental design and statistical analysis.
