# Supplementary material for: Increased accuracy of starch granule type quantification using mixture distributions
Source: Plant Methods. 2017 Dec 6;13:107. doi: 10.1186/s13007-017-0259-2 (PMC5718142; doi:10.1186/s13007-017-0259-2)
Supplement: Supplementary file 1 — Additional file 1. Experimental design and statistical analysis. [file 13007_2017_259_MOESM1_ESM.pdf]

METHODOLOGY

Supplementary information: method to quantify population of starch granule types

Emi Tanaka[1*], Jean-Phillippe F Ral[2], Sean Li[2], Raj Gaire[2], Colin R Cavanagh[3], Brian R Cullis[1] and Alex Whan[2]

Experimental Design

The trait of interest in our experiment is the proportion of the granule types. This is estimated from the granule size distribution using the two methods described in the main paper, namely the size threshold measure and mixture measure. The distribution of the granule size is obtained from a multi-phase experiment. Specifically, the experiment involved four phases: grain samples taken from the field trial (Phase I); then milled to flour (Phase II); processed further for extraction in the laboratory (Phase III); and the run through Mastersizer (Phase IV).

The treatment for our experiment is the genotype. To distinguish the sources of variation from genetic sources of variation, we require a separate randomisation and replication for each phase and an appropriate analysis that takes into account the blocking structures for each phase [1? –3]. The details of each phase are given below and factors that will be used to specify the model later are given in `this font`.

Phase I: Field trial

The field trial was arranged in a rectangular array of 81 rows by 20 columns, i.e. a total of 1620 plots. There were a total of 1077 genotypes sown. The trial was designed using the DiGGer software [4] for a p-replicate design [5] in which a proportion p of lines were sown with two plots each and the remaining with a single plot each. The 14 check genotypes had additional 3-7 plots each. The value of p is 0.48 (521 out of 1077 genotypes). The trial had 3 near-resolvable blocks, coinciding with the bay (in the sense that replicated genotypes, excluding checks, appeared at most once in each block), which were aligned with the rows such that the first replicated block comprised of rows 1-27, the second rows 28-54, and the third rows 55-81.

The pEUs are the 1620 physical locations within the field indexed by the rows and columns. The pOUs are the 1620 grain samples from these plots. The pRUs are the 1042 packets of seeds. Of the 1620 pOUs, 705 pOUs are used for the next phase of which 215 pOUs were relevant to the final phase of our experiment.

The pIU in the next phase consisted of a uniform mixture of grain samples from 2 plots or of a single plot. Each Phase II pIU (and thus pEU) is indexed by two factors, `Leftplot` and `Rightplot`, corresponding to the plot number (1-1620) and these two factors differ where the unit is a mixture of two different plots, otherwise, have the same level. Similar factors are defined for column (1-20), row (1-81) and bay (1-3) numbers (`Leftcol`, `Rightcol`, `Leftrow`, `Rightrow`, `Leftbay`, and `Rightbay`).

Phase II: Milling

In the milling phase, 10 grain samples are milled for each of the 62 days (`MillDay`) resulting in a total of 620 milled samples. These 620 milled samples are the Phase II pOUs. The pEUs are the “positions” associated with the milling run and is indexed by milling day and order. Note that `MillOrder` consists of 10 levels and is nested within `MillDay`. The grain samples were either a composite from two plots sown with the same genotype; from a single plot or split from a single plot to serve as a replicate in Phase II - we refer these grain samples as composite, single or duplicate samples, respectively, and these comprise the 620 pIUs. Specifically, the grain samples were obtained from the 705 plots from the previous phase and consisted of 131 composite samples, 397 single samples and 92 duplicate samples. Of the 397 single samples, 132 have field replicates. In total there were 508 genotypes in this phase. The milling samples are replicated such that we have two replicate blocks (`MillRep`) that coincides with the milling day. Specifically, milling replicate 1 is from milling day 1 to 31 inclusive and milling replicate 2 is from milling day 32 to 62 inclusive. The pRUs are the 92 grain samples. From 620 milled samples, 180 are used for the extraction phase.

Phase III: Extraction

In the extraction phase, the 180 milled samples (pIUs) consisting of 155 genotypes from Phase II, of which 44 milled samples were split to form as a replicate

*Correspondence: etanaka@uow.edu.au
[1]School of Mathematics and Applied Statistics, University of Wollongong, Northfields Ave, 2522 Wollongong, Australia
Full list of author information is available at the end of the article
†Equal contributor

(thus there were 88 pRUs), were then treated as described in the Methods in the main paper. In total, there were 224 milled samples prepared for extractions with 32 extractions (`ExtractOrder`) per day over 7 days (`ExtractDay`). Here `ExtractOrder` consists of 32 levels and is nested within `ExtractDay`. The pEUs are the positions corresponding to the extraction indexed by extraction day and order and the pOUs are the 224 extractions. About 21.1% (38) of the 180 milled samples were milling of the composite grain samples.

Phase IV: Mastersizer

All of the 224 extractions (Phase IV pIUs) from the previous phase, each with a replicate except for 16 extractions, was then run through the Mastersizer with 36 runs (`MSOrder`) per day for each of the 12 days (`MSDay`). Each run consisted of 2 technical replicates (`MSTecRep`) resulting in 864 particle size distribution. Thus the pOUs are these 864 particle size distribution; the OU is the technical run associated with each pOUs; and the 416 extractions are the pRU. The pEUs are the positions of the Mastersizer run indexed by day and order.

Statistical Analysis

Statistical Model

We consider the analysis of the proportion of the granule types separately in an univariate model. Let $\boldsymbol{p}_A$, $\boldsymbol{p}_B$ and $\boldsymbol{p}_C$ be the $n \times 1$ vectors of estimates of the proportion of A-type, B-type and C-type granules with each ordered by the observational units. Note that we expect the sum of the corresponding elements of $\boldsymbol{p}_A$, $\boldsymbol{p}_B$ and $\boldsymbol{p}_C$ to be less than or equal to 1, with most cases holding the equality. This is because $\boldsymbol{p}_A$, $\boldsymbol{p}_B$ and $\boldsymbol{p}_C$ are estimates which may result in rounding errors and also these proportions may not account for all the granule types measured. Let k denote the number of phases in the experiment.

We assume that there were m_d genotypes grown in the trial with $m \leq m_d$ genotypes that were used in the final phase of the experiment.

For each granule type $T \in \{\mathrm{A}, \mathrm{B}, \mathrm{C}\}$, we can write the general linear mixed model as

$$\boldsymbol{y}_T = \boldsymbol{Z}_g \boldsymbol{u}_g + \sum_{r=1}^{k} \boldsymbol{X}_r \boldsymbol{\tau}_r + \sum_{r=1}^{k} \boldsymbol{Z}_{p_r} \boldsymbol{u}_{p_r} + \boldsymbol{e} \qquad (1)$$

where $\boldsymbol{y}_T$ is the $n \times 1$ vector of the logit transformation of $\boldsymbol{p}_T$, the proportion estimate of granule type T; $\boldsymbol{u}_g$ is the $m \times 1$ vector of total genetic effects with associated design matrix $\boldsymbol{Z}_g$; $\boldsymbol{\tau}_r$ is the vector of fixed effects associated with phase r with corresponding design matrix $\boldsymbol{X}_r$; $\boldsymbol{u}_{p_r}$ is the vector of random peripheral effects associated with phase r with corresponding design matrix $\boldsymbol{Z}_{p_r}$; and $\boldsymbol{e}$ is the $n \times 1$ vector of residuals. The vectors of random peripheral effects will typically contain sub-vectors that will be denoted by $\boldsymbol{u}_{p_{rs}}$. We assume that these sub-vectors, $\boldsymbol{u}_g$ and $\boldsymbol{e}$ are jointly Gaussian distributed and mutually independent with variances given as $\mathrm{var}(\boldsymbol{u}_g) = \boldsymbol{G}_g$, $\mathrm{var}(\boldsymbol{u}_{p_{rs}}) = \boldsymbol{G}_{p_{rs}}$ and $\mathrm{var}(\boldsymbol{e}) = \boldsymbol{R}$. In the simplest model, we assume that $\boldsymbol{G}_{p_{rs}} = \sigma^2_{p_{rs}} \boldsymbol{I}$ with the dimensions of the identity matrix commensurate with the length of the corresponding vector of effects although we can include more complex forms such as a separable auto-regressive process of order 1 for the modelling of spatial correlations in the field plot effects [6, 7]. Note here that the above design matrices are the same regardless of the granule type although the corresponding effects as well as residuals are granule type dependant, we remove the subscript T for brevity.

The experimental units in the second phase include composite mixtures of the observational units in the first phase, as such the design matrix $\boldsymbol{X}_1$ and $\boldsymbol{Z}_1$ in model (1) have non-standard formats. We can rewrite $\boldsymbol{X}_1 = \boldsymbol{C}_1 \boldsymbol{X}_1^*$ and $\boldsymbol{Z}_{p_1} = \boldsymbol{C}_1 \boldsymbol{Z}_{p_1}^*$ where $\boldsymbol{C}_1$ is a matrix such that $\boldsymbol{C}_1 \boldsymbol{X}_1^* \boldsymbol{\tau}_1$ and $\boldsymbol{C}_1 \boldsymbol{Z}_1^* \boldsymbol{u}_1$ results in a vector containing averages of the associated Phase I effects over the same level of Phase II pEU. See [**?**] for examples of the $\boldsymbol{C}_1$ matrix.

Reliability calculation

We compute the reliability as the mean of the squared accuracy of each genotype [**?**]. Specifically, the accuracy of the i-th genotype for a particular granule type is the correlation of the corresponding total genetic effect to its predicted value and given as

$$r_i = \sqrt{1 - \frac{C^{gg}_{ii}}{G_{g_{ii}}}} \qquad (2)$$

where C^{gg}_{ii} is the i-th diagonal element of the prediction error variance matrix for $\boldsymbol{u}_g$ in model (1) and $G_{g_{ii}}$ is the i-th diagonal element of $\boldsymbol{G_g}$. Then the (mean-genotype) reliability is given as

$$r^2 = \frac{1}{m} \sum_{i=1}^{m} r_i^2.$$

Analysis of data

All analysis in this paper were fitted using ASReml-R package [8] within the R statistical environment [9] which uses the average information algorithm [7] for the residual maximum likelihood estimates (REML) of the variance parameters. The estimates and prediction of the fixed and random effects are then obtained by

Table 1 The number of phase-related units and treatment of the experiment.

Unit	Phase I	Phase II	Phase III	Phase IV
Treatment	1077 genotypes	508 genotypes	155 genotypes	155 genotypes
pRU	521 × 2 packets of seed	46 × 2 grain samples	44 × 2 flour samples	208 × 2 extractions
pIU	1620 packets of seed	620 grain samples	224 flour samples	432 extractions
pEU	1620 plots	620 milling runs	224 extraction runs	432 analyser runs
pOU	1620 grain samples	620 flour samples	224 extractions	864 size distribution
OU	-	-	-	864 analyser technical runs

Table 2 The REML estimation of the variance components and the mean-line reliability from the univariate models for data vector given by Method 1 (size-threshold) or 2 (mixture distribution) from the main article. Note that Phase I-IV residuals are indexed by `Plot` (`LeftPlot` and `RightPlot` in the model), `MillDay:MillOrder`, `ExtractDay:ExtractOrd`, and `MSDay:MSOrd:MSTecRep`, respectively.

	Method 1			Method 2		
Source	A	B	C	A	B	C
Geno	0.0138	0.0139	0.00795	0.0489	0.0528	0.00229
**Phase IV**						
MSDay	0.00301	0.00292	0.00164	0.007	0.00741	0.000557
MSDay:MSOrd	0.0606	0.0588	0.0225	0.147	0.135	0.0746
**Phase III**						
ExtractDay	0.0209	0.0213	0.00226	0.0386	0.0465	4.43e-10
**Phase II**						
MillRep	1.74e-10	1.24e-10	6.1e-10	3.04e-10	1.96e-10	4.43e-10
MillDay	4.49e-11	4.76e-11	1.06e-09	1.18e-10	1.7e-10	1.98e-10
**Phase I**						
Bay	0.00133	0.00142	3.83e-10	0.00283	0.00323	7e-09
Column	6.88e-09	9.13e-09	3.1e-09	1.6e-08	2.02e-08	7e-09
Row	0.00945	0.00921	0.00217	0.0117	0.0117	0.00213
Phase I Residual	1.24e-08	1.29e-08	2.98e-10	2.76e-08	4.08e-07	2.88e-10
Phase II Residual	8.61e-11	8.07e-11	1.69e-10	1.83e-10	1.55e-10	4.88e-08
Phase III Residual	0.0501	0.0492	0.0116	0.0923	0.0985	0.0461
Residual	0.000109	7.73e-05	0.000381	0.00019	0.000123	0.000277
Reliability	0.171	0.174	0.293	0.264	0.279	0.036

using these variance parameter estimates in a solution of the mixed model equations [10].

Following [1] and [2], a linear mixed model was fitted to each proportion of the granule type estimated by either the size threshold or mixing probabilities as outlined in the main paper. The fixed effects incorporated the overall mean and the check genotypes and the random effects incorporated blocking structures employed in the multiphase experiment and the genotype effect. We assume that $\boldsymbol{R} = \sigma^2 \boldsymbol{I}_n$ and $\boldsymbol{G}_g = \sigma_g^2 \boldsymbol{I}_m$. All variance-covariance matrices of the blocking terms are also assumed to be scaled identity. The syntax in ASReml-R for the random part of the model is given as

```
Random=~ Geno +
Leftbay:zero + and(Leftbay, 0.5) + and(Rightbay, 0.5) +
Leftcol:zero + and(Leftcol, 0.5) + and(Rightcol, 0.5) +
Leftrow:zero + and(Leftrow, 0.5) + and(Rightrow, 0.5) +
Leftplot:zero + and(Leftplot, 0.5) + and(Rightplot, 0.5) +
MillRep + MillDay + MillDay:MillOrder +
ExtractDay + ExtractDay:ExtractOrd +
MS.Day + MS.Day:MS.Ord
```

where the colon operator indicates an interaction term, the factor `Geno` represents the genotype effects and other factors are as defined before. Here `zero` is a vector of zeroes used for initialising the corresponding structure for computational purposes and the collective syntax `and(Leftxxx, 0.5) + and(Rightxxx, 0.5)`, where `xxx` is `row`, `col`, `bay` and `plot`, builds the non-standard design matrix $\boldsymbol{C}_1\boldsymbol{Z}_1^*$.

The estimates of the variance components and the mean-genotype reliability are shown in Table 2. It is worth noting that the REML estimate of the genetic variance for C-type granule estimated by Method 2 is very low (0.00229) - futher discussion on this matter is elaborated in the main paper.

It would be reasonable to assume the proportions of each granule type of the same sample are correlated traits. The accuracy of genotypic effects would then benefit from using a multivariate linear mixed model approach over a univariate model as above (1) [11]. In this case however, $\boldsymbol{p}_C \approx \boldsymbol{1}_n - \boldsymbol{p}_A - \boldsymbol{p}_B$ (with inequality resulting mostly due to rounding errors or in the case of mixing weights, particle size distribution fitted with four or five Gaussian mixture distribution), as such the third proportion is almost perfectly explained by the linear combination of the other two proportions. This would necessitate only fitting a bivariate model to two

sets of proportions of granule type however as shown in Figure 1, the genetic effects for the proportions of A-type and B-type granules are almost perfectly negatively correlated. We thus did not proceed with fitting a bivariate model.

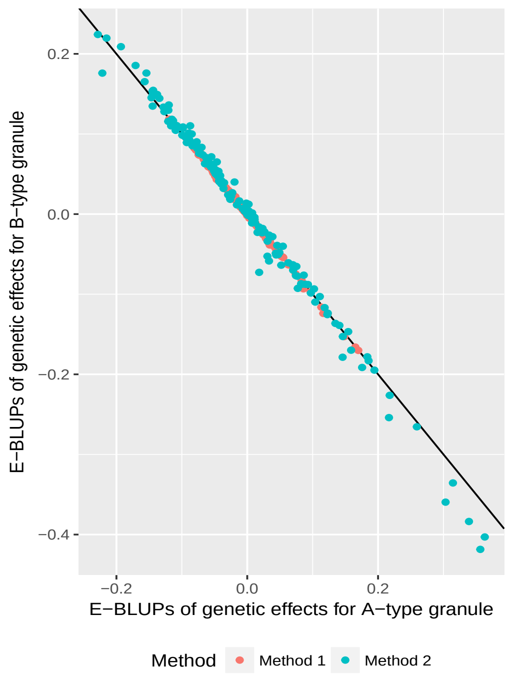

Figure 1 The plot of E-BLUPs of genetic effects in model (1) for proportion of type-A vs. type-B as estimated by Method 1 (size-threshold) or Method 2 (mixture-measure). The diagonal black line corresponds to $y = -x$.

Author details

[1]School of Mathematics and Applied Statistics, University of Wollongong, Northfields Ave, 2522 Wollongong, Australia. [2]CSIRO Agriculture and Food, 1600 Clunies Ross Street, 2601 Canberra, Australia. [3]Bayer CropScience Pty Ltd, Seeds, , Australia.

References

1. Brien, C.J., Bailey, R.A.: Multiple randomizations. Journal of the Royal Statistical Society. Series B: Statistical Methodology **68**(4), 571–609 (2006). doi:10.1111/j.1467-9868.2006.00557.x
2. Smith, A.B., Lim, P., Cullis, B.R.: The design and analysis of multi-phase plant breeding experiments. The Journal of Agricultural Science **144**(05), 393 (2006). doi:10.1017/S0021859606006319
3. Smith, A.B., Thompson, R., Butler, D.G., Cullis, B.R.: The design and analysis of variety trials using mixtures of composite and individual plot samples. Journal of the Royal Statistical Society: Series C (Applied Statistics) **60**(3), 437–455 (2011). doi:10.1111/j.1467-9876.2010.00755.x
4. Coombes, N.E.: The Reactive TABU Search for Efficient Correlated Experimental Designs. PhD thesis, Liverpool John Moores University (2002)
5. Cullis, B.R., Smith, A.B., Coombes, N.E.: On the design of early generation variety trials with correlated data. Journal of Agricultural, Biological, and Environmental Statistics **11**(4), 381–393 (2006). doi:10.1198/108571106X154443
6. Cullis, B.R., Gleeson, A.C.: Spatial Analysis of Field Experiments-An Extension to Two Dimensions. Biometrics **47**(4), 1449–1460 (1991). doi:10.2307/2532398
7. Gilmour, A.R., Cullis, B.R., Verbyla, A.P.: Accounting for Natural and Extraneous Variation in the Analysis of Field Experiments. Journal of Agricultural, Biological, and Environmental Statistics **2**(3), 269–293 (1997). doi:10.2307/1400446
8. Butler, D.G., Cullis, B.R., Gilmour, A.R., Gogel, B.J.: Mixed models for S language environments ASReml-R reference manual (2009)
9. R Development Core Team: R: A Language and Environment for Statistical Computing. R Foundation for Statistical Computing (2008)
10. Henderson, C.R.: Best linear unbiased estimation and prediction under a selection model. Biometrics **31**(2), 423–447 (1975). doi:10.2307/2529430
11. Ganesalingam, A., Smith, A.B., Beeck, C.P., Cowling, W.A., Thompson, R., Cullis, B.R.: A bivariate mixed model approach for the analysis of plant survival data. Euphytica **190**(3), 371–383 (2013). doi:10.1007/s10681-012-0791-0
